# Production of Low-Cost Adsorbents within a Circular Economy Approach: Use of Spruce Sawdust Pretreated with Desalination Brine to Adsorb Methylene Blue

**DOI:** 10.3390/ma17174317

**Published:** 2024-08-30

**Authors:** Dorothea Politi, Elias Sakellis, Dimitrios Sidiras

**Affiliations:** 1 Laboratory of Simulation of Industrial Processes, Department of Industrial Management and Technology, School of Maritime and Industrial Studies, University of Piraeus, 80 Karaoli & Dimitriou, 18534 Piraeus, Greece; doritapoliti@unipi.gr; 2 Institute of Nanoscience and Nanotechnology, National Centre for Scientific Research “Demokritos”, Agia Paraskevi Attikis, 15310 Athens, Greece; e.sakellis@inn.demokritos.gr

**Keywords:** methylene blue, adsorption, spruce, pretreatment, brine

## Abstract

A sustainable low-cost activated carbon substitute was produced based on pretreated lignocellulosic biomass, especially spruce sawdust. A harmful liquid waste, desalination brine, was used for the treatment of a solid wood industry waste, spruce sawdust. This approach is in the circular economy theory and aims at the decarbonization of the economy. Pretreated sawdust was tested as an adsorbent appropriate for the removal of a commonly used pollutant, methylene blue, from industrial wastewater. The adsorption capacity of the pretreated material was found to have increased four times compared to the untreated one in the case that the Freundlich equation was fitted to the isotherms’ data, i.e., the one with the best fit to the isotherm’s experimental data of the three isotherm models used herein. The treatment experimental conditions with desalination brine that gave maximum adsorption capacity correspond to a 1.97 combined severity factor in logarithmic form value. Moreover, a kinetic experiment was carried out with regard to the methylene blue adsorption process. The desalination brine-pretreated sawdust adsorption capacity increased approximately two times compared to the untreated one, in the case when the second-order kinetic equation was used, which had the best fit of the kinetic data of the three kinetic models used herein. In this case, the pretreatment experimental conditions that gave maximum adsorption capacity correspond to −1.049 combined severity factor in logarithmic form. Industrial scale applications can be based on the kinetic data findings, i.e., spruce sawdust optimal pretreatment conditions at 200 °C, for 25 min, with brine solution containing 98.12 g L^−1^ NaCl, as they are related to a much shorter adsorption period compared to the isotherm data.

## 1. Introduction

To purify wastewater, it is necessary to develop novel environmentally friendly technologies and inexpensive adsorbents based on renewable and available natural materials. To adsorb dyes from wastewater, new adsorbents were developed from highly available lignocellulosic biomass composed of lignin, cellulose, and hemicelluloses [1,2]. Lignocellulosic biomass can be pretreated to produce low-cost, environmentally friendly, efficient, and reusable adsorption materials [3]. Moreover, lignin makes up 10–25% of lignocellulosic biomass and is appropriate to remove dyes from wastewater effluents utilizing green techniques [4]. Numerous methods have been investigated for the removal of dyes from wastewater, including biological, chemical, and physical treatments [5]. Biological approaches make use of algae, bacteria, fungi, and isolated enzymes, acting under aerobic or anaerobic conditions, which are also cost-effective and non-toxic. On the other hand, they require specific environmental conditions for microbial growth [6].

Chemical procedures encompass processes such as oxidation, electrochemical destruction, and chemisorption. They are generally more effective than biological methods, but they necessitate specific equipment and chemical reagents; additionally, they may also produce other harmful pollutants [7]. Physical methods, like physisorption, coagulation, screening, irradiation, membrane technologies, and ion exchange, are often employed prior to biological and chemical treatments due to their simplicity and efficiency. These physical methods demonstrate high dye removal efficiency with minimal amounts of additional chemicals that are easily regenerated [8]. Physisorption is generally reversible and non-selective because it depends on weak interactions such as dipole–dipole interactions, polar interactions, hydrogen bonding, and Van der Waals forces. In contrast, chemisorption is usually irreversible and selective, depending on the formation of strong ionic bonds [9]. Among these techniques, adsorption, particularly physisorption, can be handled with less effort and has lower energy requirements, higher efficiency, and higher resistance to toxic substances. It can adsorb most dyes and generates no polluting substances [10].

Numerous adsorbents have been extensively studied for dye removal, including metal–organic frameworks [11], polymeric materials [12], clay–organic composites [13], and various carbon materials [14]. Methylene blue (MB), a dye used in this paper and the textile and leather industries, is an organic dye with a complex molecular structure. Its release into the environment poses both aesthetic and toxicological issues due to its synthetic nature, complex aromatic structure, and resistance to biodegradation [15,16,17]. Among advanced antipollution methods, adsorption is the most common, is low-cost, has a simple design, and is environmentally friendly. Adsorption involves the attachment of the adsorbate–adsorbent. The quality of adsorbents depends on their specific surface area, surface morphology, porosity, composition, multifunctional groups, and structural stability [18,19]. However, developing an ideal adsorbent capable of removal of multiple polluting substances from wastewater continues to be a substantial challenge [20]. In Table 1, we report the adsorption capacity of MB (expressed by the parameters of the Freundlich and the Langmuir isotherm equations) on various adsorbents according to some recent studies.

Saline brine from salt lakes and industrial activities has negative environmental impact. Brine from seawater desalination plants is a major environmental threat. According to Jones et al. [31] there are 15,900 energy-consuming operational desalination plants, producing 95.37 million m^3^/day of desalinated water and discharging 141.5 million m^3^/day of brine. Most of the brine is produced close to the seashore and many environmental problems are caused by the discharge of hypersaline brine into surface waters. The discharge of brine with increased salinity and toxic chemicals from the desalination process has negative ecological effects towards marine ecosystems and ocean life. The high salinity of brine increases the salinity of the nearby waters, forms ‘brine underflows’ depleting the dissolved oxygen of these waters, and can harm benthic organisms, disrupting the whole food chain [32]. Therefore, efforts are aimed at ensuring zero discharge of the brine into wastewater, as well as the reuse of this brine and its components [33]. Seawater desalination is expected to become more widespread in recent decades [34]. Brine treatment and valorization can reduce wastewater pollution and increase the amount of clean water. Minimal/zero liquid discharge desalination systems are developed for this purpose. New technologies like membrane distillation and forward osmosis are favorable for treating high-salinity brine with high recovery rates [35]. Thermal and membrane treatment technologies for desalination brine can lead to the discharge of nearly zero liquid [36]. To provide a sustainable, decarbonized (CO_2_-free) circular economy, brine valorization is necessary [37]. The development of the desalination industry and the increasing need for brine management strategies can establish adsorption as a new brine management strategy [38].

Spruce sawdust biochar (produced by pyrolysis) has been used for the adsorption of MB, copper, and cadmium from aqueous solution [39]. Untreated spruce sawdust has been used for the adsorption of heavy metals like copper [40,41], lead [40,42], cadmium, nickel, and zinc [40]. In addition, spruce sawdust was used to adsorb many dyes like Acid Orange 7, Acid Yellow 11, and Acid Red 88 [43]. Also, spruce sawdust was used to adsorb arsenic [44] and sodium salicylate [45]. Moreover, spruce sawdust was used for the effective adsorption of reactive pigments such as Reactive blue 13, Reactive blue 19, Reactive blue 4, Reactive black 5, and Reactive orange 16 [46]. On the other hand, spruce sawdust pretreated with ferrofluid was used to adsorb organic dyes such as Bismarck brown, Congo red, and Amido black [47]. Moreover, spruce sawdust pretreated with diethylene glycol and sulfuric acid was used to adsorb hexavalent chromium from wastewater [48]. In the present work, we focused on the production of an efficient ecological adsorbent based on modified spruce sawdust (lignocellulosic biomass) to be used as an inexpensive activated carbon alternative. The proposed process was the pretreatment of spruce sawdust with the desalination brine in a batch reactor. This approach follows the zero-waste circular economy theory, i.e., use a waste (desalination brine) to pretreat another waste (spruce sawdust) and then use the pretreated waste to clean a third waste (dye, e.g., methylene blue). Specifically, the solid residue of the sawdust treated with brine was utilized as an adsorbent to remove such a widely used basic dye as MB from wastewater. The suitability of the Freundlich, Langmuir, and Sips sorption isotherm equations, as well as kinetic models, for describing the MB sorption process with a modified sawdust was studied.

## 2. Materials and Methods

### 2.1. Material Development

A local wood manufacturing company provided the spruce sawdust with a humidity of 9% *w*/*w*. Material with particle sizes 0.2–1 mm was separated by screening. Spruce sawdust was 44.2% cellulose, 24.8% hemicelluloses (14.6% mannan, 6.2% xylan, 2.6% galactan and 1.4% arabinan), 28.2% acid-insoluble lignin (Klason), 0.2% ash, 2.6% extractives, and other contents (% *w*/*w* on a dry basis).

Desalination brine was initially prepared as simulated seawater according to the ASTM D 1141-98 (2021) [49] Simulated seawater consists of the following components: 24.53 g L^−1^ NaCl, 5.2 g L^−1^ MgCl_2_, 4.09 g L^−1^ Na_2_SO_4_, 1.16 g L^−1^ CaCl_2_, 0.695 g L^−1^ KCl, 0.201 g L^−1^ NaHCO_3_, 0.101 g L^−1^ KBr, 0.027 g L^−1^ H_3_BO_3_, 0.025 g L^−1^ SrCl_2_, and 0.003 g L^−1^ NaF. The concentrated brine solutions were prepared by increasing the above concentrations by 4 and 7 times. The simulated desalination brine wastewater is referred to below by giving only the concentration of NaCl due to simplicity.

### 2.2. Sawdust–Brine Treatment Process

**Figure 1:**
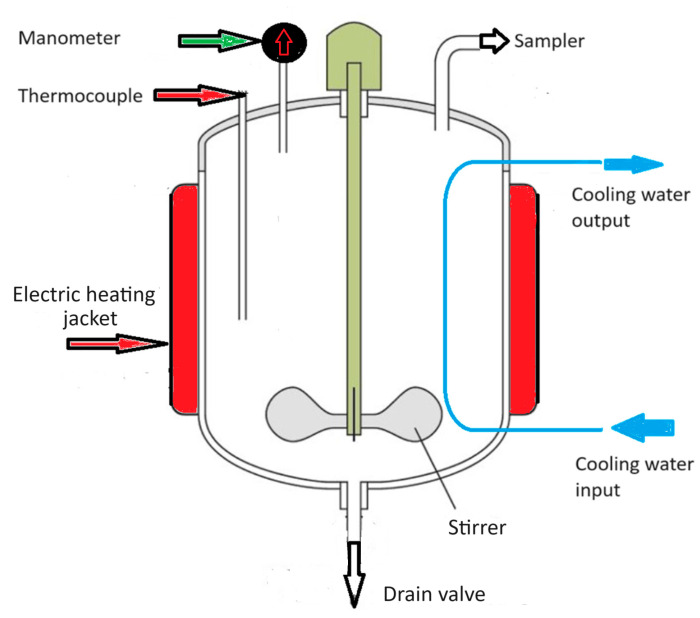
Brine pretreatment process was performed in a 4-L PARR batch reactor (autoclave).

A 4-L PARR batch reactor (autoclave) was used for the spruce sawdust with brine treatment process (see Figure 1). The isothermal sawdust–brine treatment time was 0, 25, and 50 min (to these values preheating and the cooling periods which are non-isothermal must be added). Brine and organic acids produced from the spruce sawdust through the treatment acted as catalysts. The brine solution volume in the autoclave was 1000 mL, and the sawdust quantity was 100 g, resulting in a liquid-to-solid ratio equal to 10:1. The stirring speed was 100 rpm. The temperature was 160°, 200 °C, and 240 °C and corresponds to the isothermal period’s temperature. The liquid phase was separated from the solid phase at the end of the reaction by filtering with Munktell paper sheet (grade 34/N) in a Buchner filter. Then, the solid phase was repeatedly washed with water until the complete removal of soluble substances reached a neutral pH value and dried at 110 °C for 24 h to achieve the humidity of the untreated sawdust. Then, the obtained solid particles were used as an adsorbent in experiments on the sorption of methylene blue.

### 2.3. Adsorbate

MB provided by Merck, Rahway, NJ, USA, (CI 52015: C_16_H_18_ClN3S·xH_2_O x = 2–3, molecular weight = 319.86 × 10^−3^ kg mol^−1^ anhydrous) was used as adsorbate in the adsorption isotherm and kinetic experiments. MB solution concentration was measured with a HACH DR6000 UV-VIS spectrophotometer (Hach Europe Headquarters, Dusseldorf, Germany) at λ = 664 nm. The solution pH was near 8.0 during the adsorption experiments.

### 2.4. Adsorption Isotherm Studies

Discrete batch experiments were carried for the estimation of the adsorption isotherms. The adsorbate solution was *V* = 0.5 L, the adsorbent was 0.5 g, and each experiment was carried out in 1 L bottles at 23 °C and pH = 8. The initial MB concentration was *C*_0_ = 1.6–156 mg L^−1^. The experimental system was mechanically stirred at 600 rpm for seven days, time necessary to ensure that almost equilibrium conditions are achieved. After this, a 0.1 mm nylon filter was used to separate solid from the liquid phase, i.e., the spruce sawdust adsorbent from the MB solution. Solution concentration of each bottle was measured to represent one point of each adsorption isotherm.

### 2.5. Kinetics of Adsorption

Adsorption kinetic experiments were performed in a 2 L glass reactor with stirring at 600 rpm. The aqueous phase volume was *V* = 1 L and the adsorbent mass was *m* = 1 g. The experiments took place at 23 °C. The initial MB concentration was *C*_0_ = 12 mg L^−1^. Samples of 10 mL were taken from the aqueous phase every 5 min, using a pipette. A 0.1 mm nylon filter was used to separate solid phase (sawdust) from the liquid phase (MB).

### 2.6. Experimental Design

Box–Behnken design was utilized to determine the combinations of the pretreatment variables (temperature, time, and brine concentration) [50]. This method requires a relatively small number of experiments compared to other common experimental or optimization methods [51,52]. Quantum XL (SigmaZone, Orlandi, FL, USA) software version 17, was used for the planning of the experiments. This plan is presented in Table 2.

### 2.7. Combined Severity Factor

The pretreatment time, temperature, and brine concentration effects in a single variable were incorporated into a combined severity factor (CSF), based on the one introduced by Brasch and Free [53] 1965, P-factor, for the case of the lignocellulosic biomass isothermal prehydrolysis and Kraft pulping. Later, this factor was applied by Overend and Chornet [54] in the case of fractionation of lignocellulosics and called ‘reaction ordinate’. The P-factor was:

P-factor = t·exp[(T − 100)/14.75](1)

where t is the time (in min) and T is the temperature in °C. 

Chum et al. [55] and Abatzoglou et al. [56] incorporated the pH of the acidic or alkaline liquid phase effect as follows:

R′_0_ = 10^−pH^·t·exp[(T − 100)/14.75] (2)

Various isothermal pretreatments can be simulated using the CSF. Lloyd and Wyman [57] used CSF to simulate dilute-acid pretreatment in the case of softwood and corn stover feedstock. Kabel et al. [58] used CSF to simulate wheat straw pretreatment.

In the case of desalination brine pretreatment of spruce sawdust, we will use the CSF similarly as in other non-isothermal pretreatment cases [59,60,61], as follows:

(3) R0*=10−pH∫0texp⁡(T−10014.45)dt

where pH refers to the liquor after the desalination brine pretreatment. Equation (3) is different than Equation (2), because it is expressing the variation of the pretreatment temperature over time, considering not only the isothermal period (as usual) but also the non-isothermal preheating and cooling periods.

### 2.8. Other Analytical Techniques

The quantitative saccharification method [62] was applied to all pretreated and untreated spruce sawdust samples to analyze the formed liquid phase for glucose, mannose, xylose, and arabinose. For this analysis, HPLC (Agilent 1200 equipped with Aminex HPX-87H Column, refractive index detector, and 5 mM H_2_SO_4_ in water as the mobile phase, Santa Clara, CA, USA) was used according to the high-performance liquid chromatography method. According to the quantitative saccharification method, glucan was produced from cellulose, and mannan, xylan, and arabinan was produced from the hemicelluloses. The Tappi T222 (2002) method “Acid insoluble lignin in wood and pulp” was applied for the determination of the acid-insoluble lignin (Klason lignin) of all pretreated and untreated spruce sawdust samples. A MultiLab model 540 digital pH meter (Weilheim, Germany) was utilized for the pH measurements.

The Brunauer, Emmett, and Teller (BET) method [63] assumes that gas adsorption assesses the surface area of all materials with accessible porosity, depending on the chosen adsorptive. The ISO 9277:2022 “Determination of the specific surface area of solids by gas adsorption—BET method” [64] was applied herein using liquid nitrogen (N_2_) at 77 K using a Micrometrics, Model Tristar II 3030 Plus Kr, Micrometrics, Lincolnshire, UK. A BET multi-point equation for the N_2_ adsorption–desorption isotherm was fitted to the experimental points to predict the specific surface area of the spruce sawdust untreated and pretreated samples dried by freeze-drying for 1 h before testing.

The scanning electron microscope (SEM) examination of the untreated and pretreated spruce sawdust samples was performed using a FEI INSPECT SEM equipped with an EDAX super ultra-thin window analyzer for energy dispersive X-ray spectroscopy (EDS) at the Institute of Materials Science of the National Center for Scientific Research ‘Demokritos’, Athens, Greece. 

Fourier transform infrared (FTIR) spectra of the untreated and pretreated spruce sawdust samples were performed using a spectroscope (MAGNA-IR 750 Spectrometer, Series II, Nicolet, SpectraLab, Markham, ON, Canada) at the Institute of Materials Science of the National Center for Scientific Research ‘Demokritos’. Diffuse reflectance sampling technique was used herein scanning for wavenumber 650–3500 cm^−1^ of the powder sawdust samples.

Powder X-ray diffraction (XRD) patterns of the untreated and the pretreated spruce sawdust samples were determined using a Bruker Model D2 Phaser diffractometer (Karlsruhe, Germany) with Ni-filtered Cu Kα radiation (λ = 1.54056 Å), working in θ–2θ geometry, 30 kV/10 mA, continuous scan mode and using a Plexiglas-based sample holder at room temperature. The scan range was 2θ = 5–70. Cellulose crystallinity index (CrI) was estimated with the equation CrI = (I_200_ − I_a_)/I_200_, where I₂₀₀ was the cellulose total intensity peak at 2θ = 22.4°, and Iₐ was the amorphous cellulose intensity peak at 2θ = 18.5° [65,66].

## 3. Results and Discussion

### 3.1. Severity Factor and Combined Severity Factor Calculations

**Figure 2:**
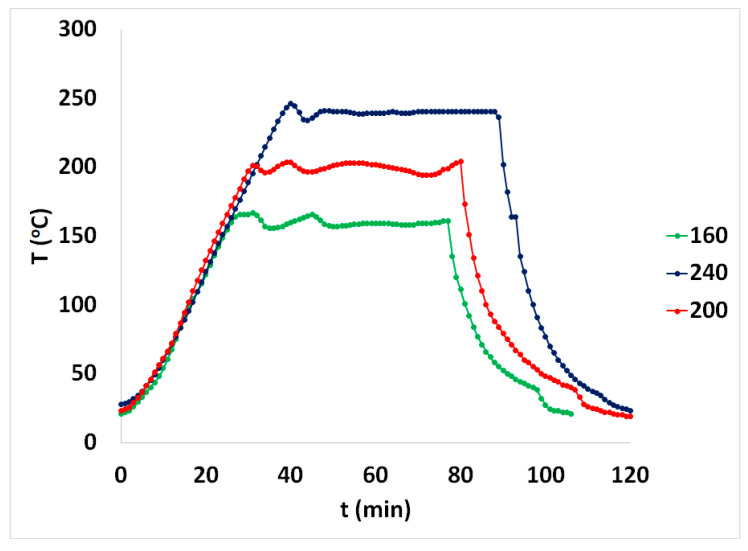
Temperature vs. time of the spruce sawdust desalination brine pretreatment experiments using a 4-L autoclave for 160, 200, and 240 °C ending temperatures.

**Figure 3:**
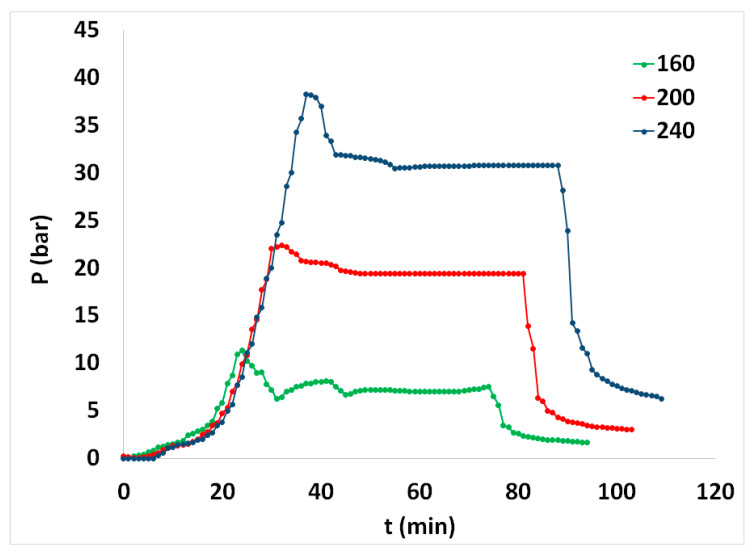
Autoclave pressure profile vs. time of the spruce sawdust desalination brine pretreatment experiments at 160, 200, and 240 °C ending temperatures.

The profile of the temperature of some of the spruce sawdust desalination brine pretreatment experiments in the autoclave is given in Figure 2, while the corresponding pressure profile is given in Figure 3 as a function of the time of the spruce sawdust desalination brine treatment batch experiments. In Figure 2, the separation of the reaction time into (i) preheating period, (ii) isothermal period, and (iii) cooling period is clearly visible.

In Table 2, the estimated values for spruce sawdust desalination brine pretreatment experiments are given regarding the severity factor *R*_0_, calculated according to Equation (3) without the factor 10^−pH^, the combined severity factor *R*_0_* calculated according to Equation (3), and the logarithm of the combined severity factor log*R*_0_*.

**Figure 4:**
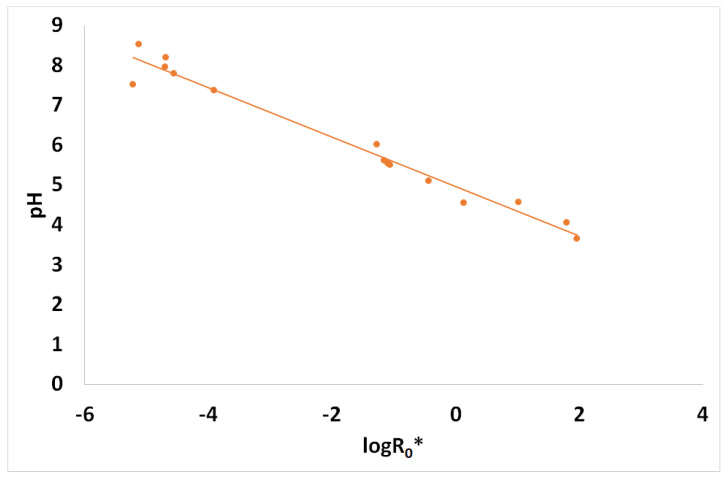
Spruce brine pretreatment’s liquid phase pH vs. the combined severity factor logarithm.

The resulting spruce sawdust desalination brine pretreatment liquid phase pH values are presented in Figure 4 vs. the logarithm of the combined severity factor which was presented in Table 3.

**Figure 5:**
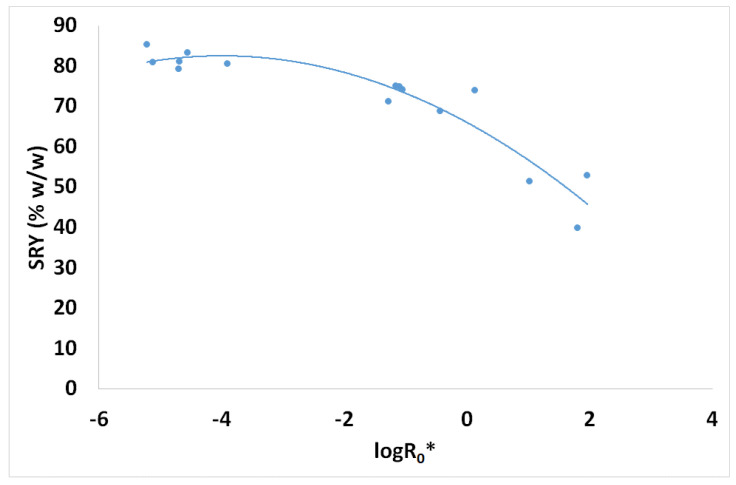
Spruce sawdust desalination brine pretreatment experiments solid residue yield (SRY) vs. the combined severity factor logarithm.

**Figure 6:**
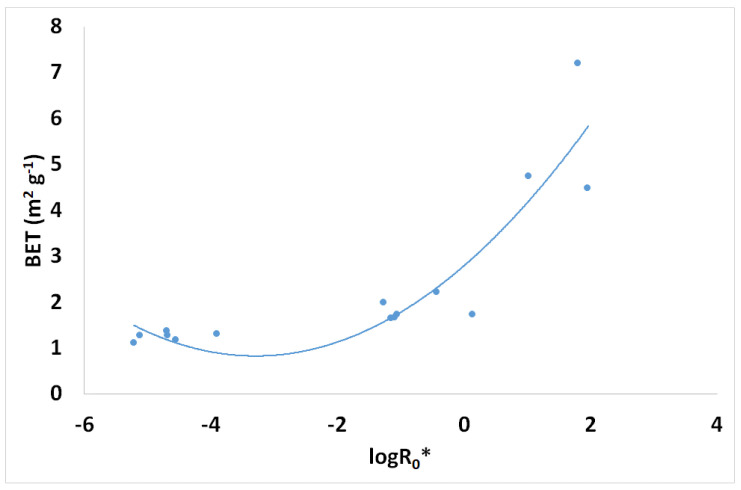
Spruce sawdust desalination brine pretreatment experiments solid residue BET values vs. the combined severity factor logarithm.

Spruce brine pretreatment’s solid residue yield (SRY) values are given in Figure 5 vs. the logarithm of the combined severity factor. Moreover, the corresponding BET values of these solid samples are also shown in Figure 6 vs. the combined severity factor logarithm. BET increases 10.2 times, i.e., from 0.703 m^2^/g for the untreated spruce sawdust to 7.19 m^2^/g for the pretreated material with CSF logR_0_* = 1.808. At the same severity pretreatment conditions, SRY reduces from 100% *w*/*w* (untreated spruce sawdust) to 39.8% *w*/*w* (pretreated material).

### 3.2. Adsorption Isotherms

Three isotherm models are analyzed: Freundlich [67], Langmuir [68], and Sips [69]. They are widely applied to fit the experimental data of the dye’s adsorption on activated carbons and untreated and pretreated lignocellulosic materials. 

The Freundlich isotherm [67] can be expressed as follows:

(4) $$q=K_{F}\times (C_{e}{)}^{\frac{1}{n}}$$

where *q* is the adsorbed amount per adsorbent mass unit (mg g^−1^), *C_e_* is the adsorbate equilibrium concentration (mg L^−1^), and *K_F_* [(mg g^−1^) (L mg^−1^)^1/n^] and *n* are constants related to adsorption capacity and adsorption intensity, respectively. Equation (4) in logarithmic form is formed as follows:

(5) $$\log q=\log K_{F}+\frac{1}{n}\log C_{e}$$

Linear and non-linear regression analysis (NLRA) was applied to calculate the *K_F_* and *n* parameter values by simulating the MB adsorption experimental data. 

The standard error of estimates (SEE) was determined as follows:

(6) $$\mathrm{SEE}=\sqrt{\sum_{i=1}^{n^{\prime }}\left(y_{i}-y_{i,theor}{)}^{2}/(n^{\prime }-p^{\prime }\right)}$$

where *y_i_* is the dependent variable experimental value, *y_i,theor_* is the theoretical value of the dependent variable, *n*’ is the experimental point number, *p*’ is the parameter number, and (*n’* − *p’*) are the degrees of freedom. 

**Figure 7:**
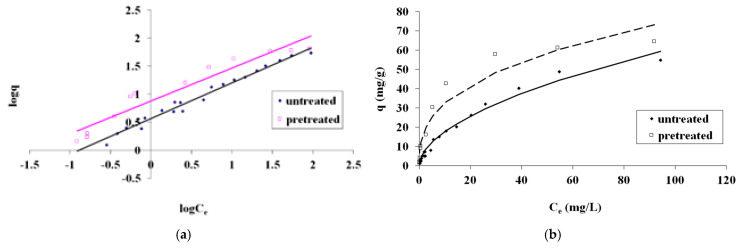
Freundlich isotherm model for methylene blue adsorption on pretreated (240 °C, 25 min, 178.71 mg/L NaCl) and untreated spruce sawdust (**a**) logq vs. logCe and (**b**) q vs. Ce. Brine concentrated seven times compared to the simulated seawater.

The Freundlich isotherm model fitting of the MB adsorption on untreated and pretreated (240 °C, 25 min, 178.71 mg/L NaCl) spruce sawdust is presented in Figure 7 as (a) *logq* vs. *logC_e_* and (b) *q* vs. *C_e_*. The pretreatment was with brine concentrated seven times compared to the simulated seawater.

The Langmuir isotherm model [68] refers to the ‘pseudo-monolayer’ adsorption theory and is formed as follows:

(7) $$q=\frac{K_{L}q_{m}C_{e}}{1+K_{L}C_{e}}$$

or

(8) $$\frac{1}{q}=\left(\frac{1}{q_{m}}\right)+\left(\frac{1}{K_{L}\cdot q_{m}}\right)\cdot \left(\frac{1}{C_{e}}\right)$$

where *K_L_* is related to the adsorption energy constant (L mg^−1^) and *q_m_* is the saturation adsorbed dye amount (mg g^−1^). These parameters can be assessed by plotting 1/*q* versus 1/*C_e_* or by NLRA. In this case, a dimensionless constant called ‘equilibrium parameter’ or ‘separation factor’ *R_L_*, can be formed with the following equation:

(9) $$R_{L}=\frac{1}{1+K_{L}\cdot C_{0}}$$

where *C*_0_ is the initial dye concentration (mg L^−1^) and *K_L_* is the Langmuir constant in L mg^−1^. Moreover, *R_L_* > 1 means unfavorable adsorption, *R_L_* = 1 means linear adsorption, 0 < *R_L_* < 1 means favorable adsorption, and *R_L_* = 0 means irreversible adsorption. 

**Figure 8:**
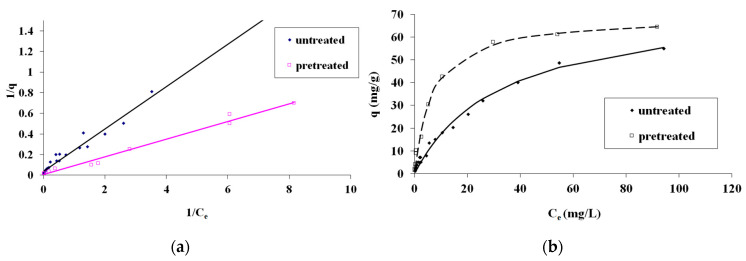
Langmuir isotherm equation for methylene blue adsorption on pretreated (240 °C, 25 min, 178.71 mg/L NaCl) and untreated spruce sawdust (**a**) 1/q vs. 1/C_e_ and (**b**) q vs. C_e_. Brine concentrated seven times compared to the simulated seawater.

In Figure 8, the Langmuir isotherm equation fitting for the MB adsorption on untreated and pretreated (240 °C, 25 min, 178.71 mg/L NaCl) spruce sawdust is presented as (a) *1/q* vs. *1/C_e_* and (b) *q* vs. *C_e_*. The pretreatment was achieved with brine concentrated seven times compared to the simulated seawater, i.e., with 178.71 mg/L NaCl and other salts.

The Sips isotherm model, also called the Langmuir–Freundlich isotherm equation [69], can be stated as follows:

(10) $$q=\frac{q_{m}\cdot {\left(K_{L}\cdot C_{e}\right)}^{1/n}}{1+{\left(K_{L}\cdot C_{e}\right)}^{1/n}}$$

where *K_L_*, *q_m_* are the Langmuir constants and *n* is the Freundlich constant. NLRA was applied to determine these parameters.

**Figure 9:**
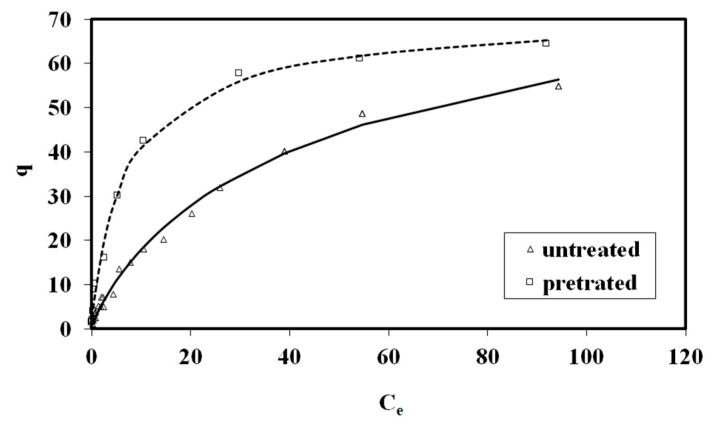
Sips isotherm model for methylene blue adsorption on pretreated (240 °C, 25 min, 178.71 mg/L NaCl) and untreated spruce sawdust, q vs. C_e_. Brine concentrated seven times compared to the simulated seawater.

In Figure 9, the Sips isotherm model for MB adsorption on pretreated (240 °C, 25 min, 178.71 mg/L NaCl) and untreated spruce sawdust is presented. The desalination brine solution was concentrated seven times compared to the simulated seawater.

**Figure 10:**
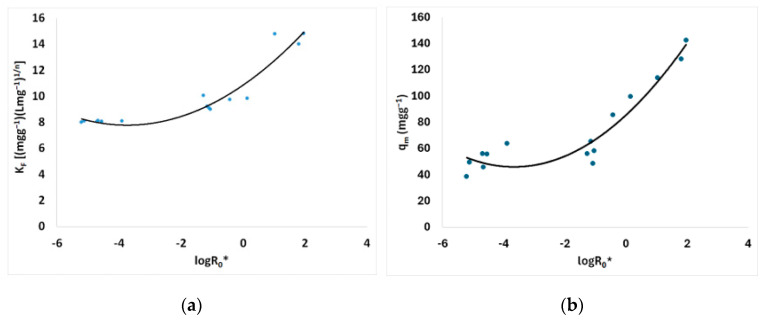
(**a**) Freundlich capacity *K_F_* and (**b**) Langmuir capacity *q_m_* parameters of the isotherm models vs. combined severity factor logarithm.

The Freundlich isotherm model’s capacity *K_F_* parameter is presented in Figure 10 vs. the logarithm of the combined severity factor. According to the capacity parameter *K_F_* value for brine-pretreated material (240 °C, 25 min, 178.71 mg/L NaCl), an increase was noticed up to 14.8 (mg g^−1^) (L mg^−1^)^1/n^ compared to that of the untreated spruce sawdust, which was 3.8 (mg g^−1^) (L mg^−1^)^1/n^.

In Table 3, the parameters of the Freundlich and the Langmuir isotherm models, as well as the SEE values, are presented. In Table 4, the parameters of the Sips isotherm model and the SEE values are given. The average SEE value for Freundlich isotherm model was lower compared with the average SEE value for the Langmuir and the Sips isotherm models, showing that among these three equations, the Freundlich equation best fits the isotherm experimental data.

The Freundlich isotherm model’s capacity *K_F_* parameter is presented in Figure 10a vs. the logarithm of the combined severity factor. According to the capacity parameter *K_F_* value for brine-pretreated material (240 °C, 25 min, 178.71 mg/L NaCl), an increase was noticed up to 14.8 (mg g^−1^) (L mg^−1^)^1/n^ compared to that of the untreated spruce sawdust, which was 3.8 (mg g^−1^) (L mg^−1^)^1/n^; i.e., it increased approximately 3.9 times. The corresponding combined severity factor (in logarithmic form) log*R*_0_* was equal to 1.964 provided the theoretically maximum capacity parameter *K_F_* value. The Langmuir isotherm model’s capacity *q_m_* parameter is presented in Figure 10b vs. the logarithm of the combined severity factor. Langmuir isotherm capacity *q_m_* for pretreated material (240 °C, 25 min, containing 178.71 g L^−1^ NaCl and other contents) increased up to 142.4 mg g^−1^ compared to that of the untreated spruce sawdust, which was 26.0 mg g^−1^; i.e., it increased approximately 5.5 times. Moreover, Sips isotherm capacity *q_m_* for pretreated material (240 °C, 25 min, containing 178.71 g L^−1^ NaCl and other contents) increased up to 72.1 mg g^−1^ compared to that of the untreated spruce sawdust, which was 36.4 mg g^−1^; i.e., it increased approximately two times (see Table 5).

**Figure 11:**
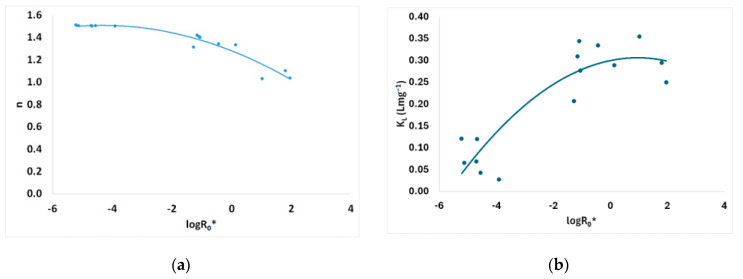
(**a**) Freundlich intensity *n* and (**b**) Langmuir intensity *K_L_* of the isotherm models vs. combined severity factor logarithm.

Moreover, the intensity *n* parameter of the Freundlich isotherm model is shown in Figure 11a vs. the logarithm of the combined severity factor. A decrease in parameter *n* translates into the increase in the power of this equation 1/*n* and consequently improvement of the adsorptivity of the desalination brine-modified spruce sawdust. The same results are supported by Langmuir parameter *K_L_*, as shown in Figure 11b.

### 3.3. Kinetics of Adsorption

The most widely used kinetic models for dye adsorption on lignocellulosics are Lagergren’ s model or the first-order kinetic model [70], second-order kinetic model [71], and intraparticle diffusion kinetic model [72]. 

The Lagergren equation [70] is as follows:

(11) $$q-q_{t}=q\cdot e^{-k\cdot t}$$

where *q* is the amount of MB adsorbed per unit mass of the adsorbent (in mg g^−1^) at equilibrium time (*t*→∞) and *q_t_* is the amount of MB adsorbed per unit mass of the adsorbent at random adsorption time *t*. Parameter *k* is the adsorption pseudo-first-order rate constant. Additionally,

(12) $$q=(C_{0}-C_{e})V/m$$

and

(13) $$q_{t}=(C_{0}-C)V/mq$$

where *C* is the concentration of MB in the bulk solution at time *t*, *C*_0_ is the concentration of MB in the solution at zero time, and *C_e_* is the concentrations of MB in the solution at ∞ time. The mass of the adsorbent (in g) is m is and the solution volume (in mL) is V. The logarithmic form of Equation (13) is:

(14) $$\ln(q-q_{t})=\ln q-k\cdot t$$

The second-order kinetic model [71] can be expressed by the equations:

(15) $$q_{t}=q-{\left[q^{-1}+k_{2}t\right]}^{-1}$$

(16) $$q_{t}=q-\frac{1}{\frac{1}{q}+k_{2}t}$$

The intraparticle diffusion model [72] can be formed as follows:

(17) $$q_{t}=c+k_{p}\cdot \sqrt{t}$$

where *q_t_* is the amount of MB adsorbed at time *t*, *c* is an empirical parameter (mg g^−1^), and *k_p_* is the intraparticle diffusion rate constant in mg g^−1^ min^−0.5^.

**Figure 12:**
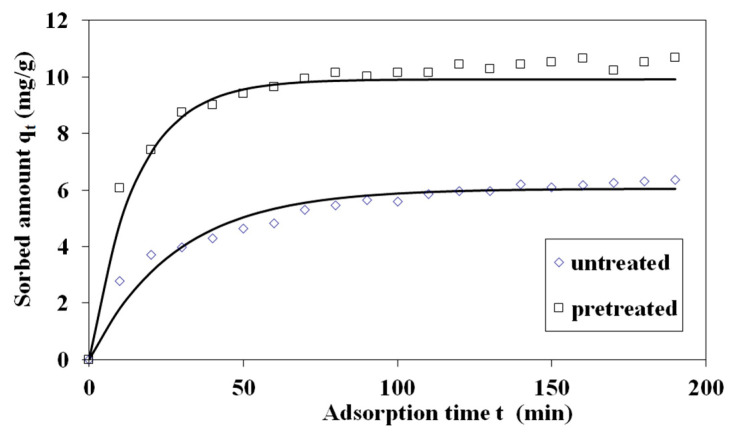
The Lagergren (first-order) kinetics of methylene blue adsorption on pretreated (200 °C, 25 min, 98.12 mg/L NaCl) and untreated spruce sawdust. Adsorption temperature 23 °C, initial methylene blue concentration C_0_ = 12 mg L^−1^, m/V = 1 g L^−1^.

**Figure 13:**
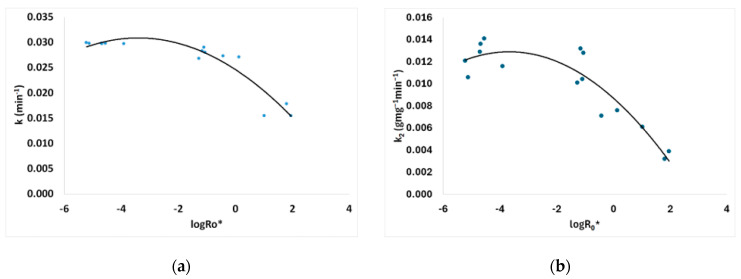
(**a**) Lagergren (first-order) and (**b**) second-order adsorption kinetic rate parameter for methylene blue adsorption on pretreated and untreated spruce vs. the logarithm of the combined severity factor.

**Figure 14:**
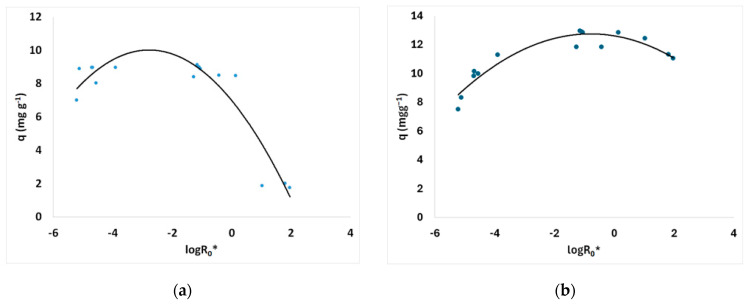
(**a**) Lagergren (first-order) and (**b**) second-order adsorption capacity parameter for methylene blue adsorption on pretreated and untreated spruce vs. the logarithm of the combined severity factor.

Pretreated and untreated sawdust first-order adsorption kinetics are presented in Figure 12. The adsorption kinetic rate parameter *k* of the Lagergren (first-order) kinetic model for MB adsorption on treated and untreated spruce sawdust vs. the logarithm of the combined severity factor is presented in Figure 13a. The Lagergren (first-order) kinetic model capacity parameter *q* for MB adsorption on pretreated and untreated spruce sawdust vs. the logarithm of the combined severity factor is shown in Figure 14b. 

**Figure 15:**
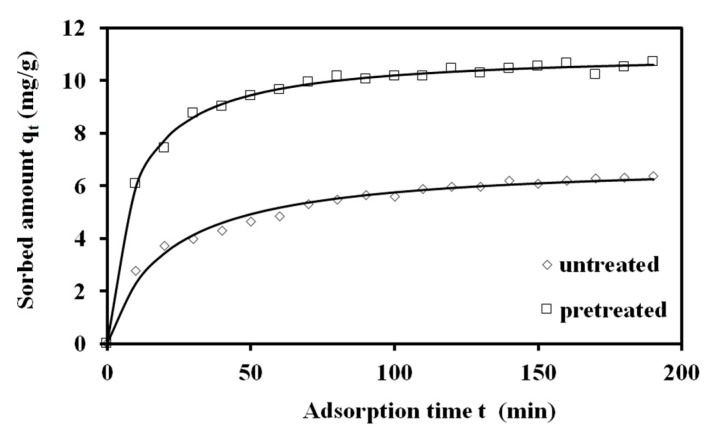
Second-order kinetics of methylene blue adsorption on pretreated (200 °C, 25 min, 98.12 mg/L NaCl), untreated, and spruce sawdust. Adsorption temperature 23 °C, methylene blue initial concentration Co = 12 mg L^−1^, m/V = 1 gL^−1^.

Second-order kinetics are given in Figure 15 for MB adsorption on pretreated and untreated spruce sawdust. The adsorption kinetic rate parameter *k*_2_ of the second order kinetic model for MB adsorption on treated and untreated spruce sawdust vs. the logarithm of the combined severity factor is presented in Figure 13b. The second-order kinetic model capacity parameter *q* for MB adsorption on pretreated and untreated spruce sawdust vs. the logarithm of the combined severity factor is shown in Figure 14b.

In Table 6 are presented parameters of the Lagergren (first-order) and the second-order kinetic models, as well as the SEE values. The average SEE value for the second-order kinetic model was lower compared with the SEE for the Lagergren kinetic model, showing that the second-order equation fits the experimental data of the MB adsorption better.

**Figure 16:**
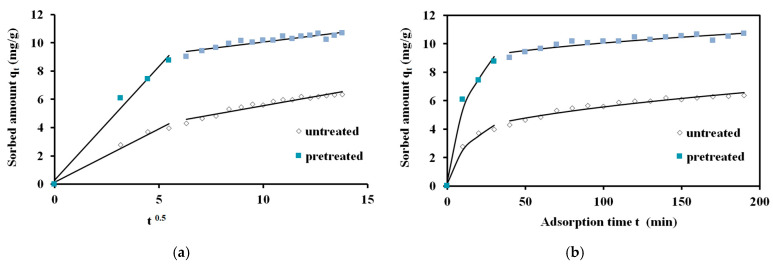
Intraparticle kinetics of methylene blue adsorption on untreated and brine-treated (200 °C, 25 min, 98.12 NaCl) spruce sawdust (**a**) *q_t_* vs. *t*^0.5^ and (**b**) *q_t_* vs. *t*. Adsorption temperature 23 °C, MB initial concentration C_0_ = 12 mg L^−1^, m/V = 1 gL^−1^.

The intraparticle diffusion model shown in Figure 16 confirms the existence of intraparticle diffusion. This means a multistage adsorption, including mass transfer of adsorbate molecules to the adsorbent external surface and then mass transfer to the internal adsorbent surface and finally adsorbate molecules sorption on the adsorbent active sites [46].

In Table 7, the parameters of the intraparticle diffusion kinetic model and SEE values are given. The brine pretreatment of the spruce sawdust at the optimal conditions (200 °C, 25 min, 98.12 NaCl) achieved an increase by 2.2. times for the adsorption rate constant *k_p_* of the first step compared to that for the untreated sawdust. The increase in the *k_p_* of the second step for the pretreated sawdust was not significant compared to that for the untreated sawdust. The average SEE value for the intraparticle diffusion model was lower compared with the SEE for the Lagergren kinetic model but higher than the average SEE for the second-order kinetic model.

The intraparticle diffusion model can find the rate that determines the diffusion mechanism. Equation (17) expresses the Webber’s pore diffusion model. Intraparticle diffusion was the rate limiting step because *q* versus *t*^0.5^ was linear, but the intercept c was not equal to zero. This means that film diffusion, along with intraparticle diffusion, probably is present. According to Figure 16, two steps define the adsorption. Probably the first part of the line relates to film diffusion, while the second part relates to inside adsorbent diffusion. External mass transfer relates to the first part of the line, while intraparticle diffusion or pore diffusion relates to the second part [73,74].

SEE values for the second-order model were decreased compared to those of the first-order kinetic model and the intraparticle diffusion model. This fact indicates the better fitting of the second-order kinetic equation to the adsorption of MB on untreated and pretreated spruce sawdust.

Adsorption is an appropriate technique for elimination of dyes from wastewater and cleaning of polluted wastewater groundwater and surface water [75]. Forest or agricultural waste-based sorbents, like desalination brine-pretreated spruce sawdust, have increased adsorption capacities, especially in the case of dyes. These sorbents have analogous adsorption capacities to the conventional commercial adsorbents like activated carbon. The pretreatment improves the dye’s adsorption capacity. Nevertheless, there is still research to be performed for novel, cost-effective, multi-function adsorbents before replacing the traditional adsorbents [76]. Adsorbents like desalination brine-pretreated spruce sawdust can be proved low cost with high efficiency, regeneration capability, and low environmental impact.

### 3.4. SEM, FTIR, and XRD of Untreated and Modified Spruce Sawdust

**Figure 17:**
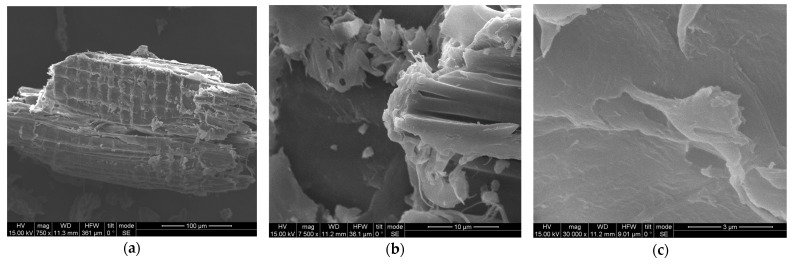
SEM of untreated spruce sawdust with magnification (**a**) 750×, (**b**) 7500×, and (**c**) 30,000×.

**Figure 18:**
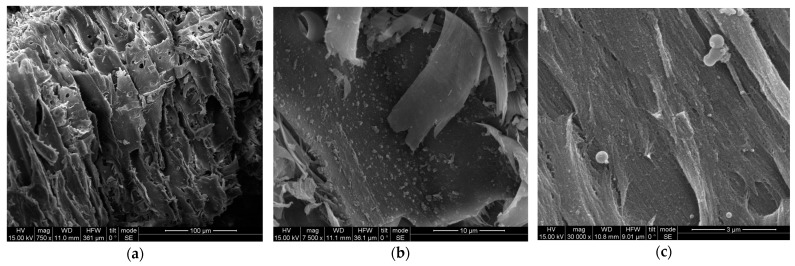
SEM of pretreated spruce sawdust. The pretreatment was using desalination brine (containing 178.71 g L^−1^ NaCl and other contents) at 200 °C for 50 min. The magnification was (**a**) 750×, (**b**) 7500×, and (**c**) 30,000×.

Untreated spruce sawdust SEM micrographs are shown in Figure 17, while Figure 18 shows those of the pretreated one using desalination brine containing 178.71 g L^−1^ NaCl and other contents at 200 °C for 50 min, with magnification (a) 750×, (b) 7500×, and (c) 30,000×. The greater roughness of the surface of the pretreated sawdust compared to the untreated spruce is more obvious at 30,000× magnification. The roughness of the brine-treated material surface facilitates the adsorption of MB, resulting in capacity values, *K_F_*, enhanced up to four times. According to the literature, numerous other physical and chemical pretreatments significantly affect the lignocellulosic material walls. The spruce sawdust surface is smooth and compacted before pretreatment, but afterwards it is loose, separated, and broken. The present results support previous reports that pretreatment techniques have a considerable impact on the structural configuration of the lignocellulosic materials [77]. The morphological changes in the cell wall structure of untreated and pretreated sawdust are clearly demonstrated in the SEM images. Lignin coating over cellulose and hemicellulose fibers makes the surface formation of untreated sawdust smooth, compact, rigid, and highly ordered. Lignin and hemicelluloses degradation disrupts the cell surface [78]. Untreated spruce sawdust exhibits a complex structure with highly fibrillar morphology of strongly clustered wood fragments. Rougher, irregular, and more porous particles were produced due to the pretreatment [79].

**Figure 19:**
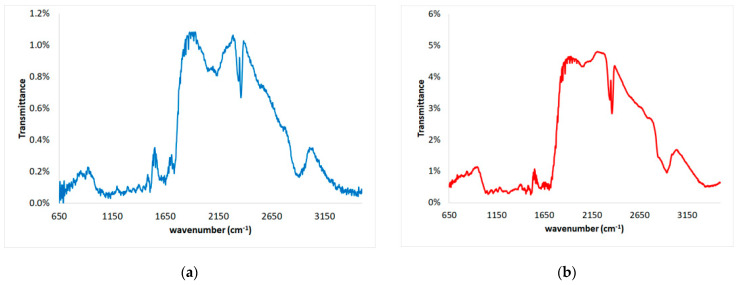
FTIR spectra of (**a**) untreated and (**b**) desalination brine-pretreated spruce sawdust.

In Figure 19, the FTIR spectra of untreated and pretreated spruce sawdust is presented while in Table 8 the corresponding peaks of these FTIR spectra and their assignment as regards untreated and pretreated spruce sawdust are given. It can be mentioned that the peak at wavenumber 3465 cm^−1^ of the untreated spruce, allocated to O-H stretching of bonded hydroxyl groups, decreased by 115 units due to cellulose/hemicellulose/lignin changes during the desalination brine pretreatment process. On the other hand, the peat at 2910 cm^−1^ of the untreated spruce, allocated to symmetric aromatic methoxyl groups and in methyl and methylene groups of side chains C-H stretching, increased by 30 units due to the lignocellulosic complex changes during pretreatment. Hemicellulose decomposition during pretreatment is responsible is responsible for the decrease in the peak at 1735 cm^−1^, assigned to C=O stretching in unconjugated xylans, to 1710 cm^−1^. Moreover, structural changes in lignin explain the increase in the peak at 1435 cm^−1^, appointed to C-H deformation in methyl and methylene, by 22 units. The peak at 1335 cm^−1^ decreases by 21 units and is assigned to spruce cellulose, hemicellulose, and lignin-C5 substituted aromatic units, C-O stretching, and CH_2_ wagging. In addition, the peak at 1042 cm^−1^ increased by 17 units and is assigned to the lignocellulosic matrix C-OH stretching vibration and C-O deformation. Finally, the 902 cm^−1^ peak allocated to C-O-C stretching decreased 35 units due to cellulose/hemicelluloses degradation, while the 805 cm^−1^ peak assigned to C-H aromatic out-of-plane bending, increased by 47 units due to the lignin structure modification during the pretreatment with brine. These findings are comparable to FTIR analysis findings by numerus researchers regarding chemical changes in wood induced by various pretreatments [80,81,82].

**Figure 20:**
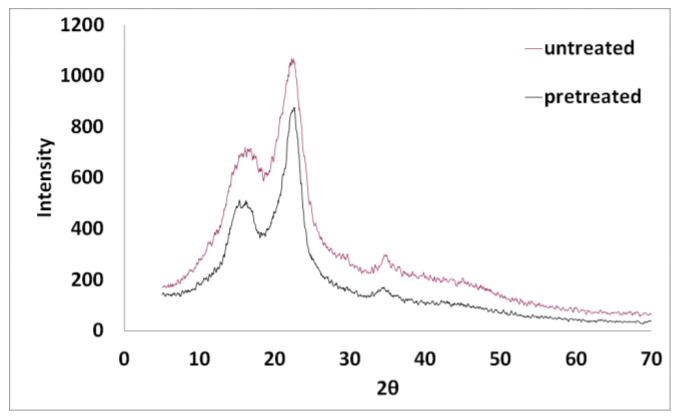
XRD patterns of desalination brine-pretreated and untreated spruce sawdust. The pretreatment was using desalination brine (containing 178.71 g L^−1^ NaCl and other contents) at 200 °C for 50 min.

**Figure 21:**
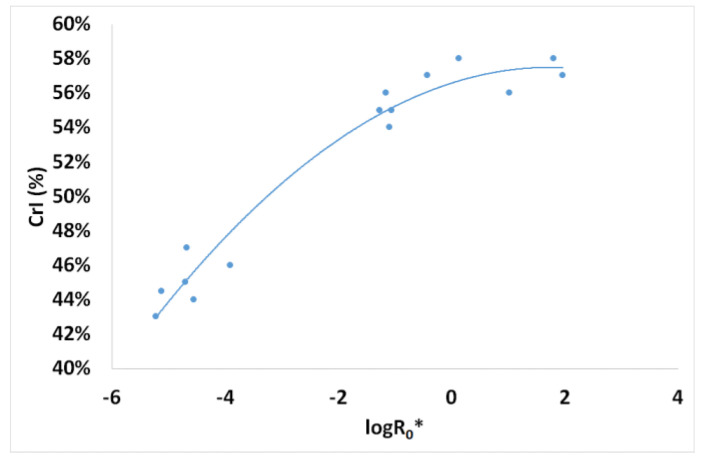
Crystallinity index (CrI) of desalination brine-pretreated spruce sawdust vs. the logarithm of the combined severity factor (log*R*_0_*).

In Figure 20 are shown the XRD patterns of untreated and pretreated spruce sawdust. The pretreatment was using desalination brine (containing 178.71 g L^−1^ NaCl and other contents) at 200 °C for 50 min. The XRD pattern peaks were noticed at 2θ of approximately 22.4° for crystalline cellulose and 18.5°for amorphous cellulose. The effect on the crystallinity of desalination brine-pretreated spruce sawdust was expressed by the crystallinity index, CrI, which increased from 44% for the untreated sawdust to 58% for the above-mentioned pretreated material. In general, the pretreated materials had CrI increasing from 44% to 58% due to the resistance of the crystalline fraction to the brine pretreatment, as presented in Figure 21. XRD examines the microstructure of cellulose in the material by diffracting and forming graphic patterns in crystalline cellulose. Lignocellulosic materials are composed crystalline (cellulose) and non-crystalline or amorphous (hemicellulose and lignin) structures. The principal peak was detected at approximately 22.40° for 2θ. The cellulose crystallinity of the lignocellulosic materials can be affected by the various pretreatment methods used [77,78,83,84].

### 3.5. Discussion on Scalability and Limitations

The application of this study, after further investigation, could help to develop a zero-waste circular economy. In this approach, desalination brine (a common waste of the desalination industry) is used instead of acids (sulfuric acid, phosphoric acid, and other contents) to pretreat spruce sawdust (a common waste of the furniture manufacturing industry and generally from the wood manufacturing industrial sector). So, no pure chemicals are used for the process. Finally, the solid residue of the sawdust pretreated with desalination brine was utilized as an eco-friendly adsorbent to remove pollutants from the aquatic environment, and, as an example, MB (a widely used basic dye) was used as a wastewater pollutant.

In the case of using argan nutshell sawdust with merchant-grade phosphoric acid to adsorb uranium and other heavy metals, Qamouche et al. [85] suggested, besides extracting toxic heavy metals, the co-extraction of other valuable materials like rare-earth elements. Moreover, they proposed an integrated utilization pathway where the used adsorbent can be used to produce biogas or energy production. The extracted metals can be recovered from the remaining ash after gasification. In this work, the brine treated spruce sawdust can be similarly used for biogas and energy production after its application as adsorbent. On the other hand, it could be used to absorb substances other than MB, e.g., other dyes or heavy metals. This approach requires further investigation. 

Similarly, Slavov et al. [86] has presented the possible approaches to the valorization of rose waste biomass. There are common methods of disposal such as discarding, composting, and use for animal food. On the other hand, there may be still-pending investigations of other promising methods for utilization, such as aroma extraction and biologically active substance recovery. Moreover, novel approaches investigate polysaccharide extraction from the waste biomass and integrated methods for biomass holistic valorization. In the concept of the integrated valorization, our work can have a follow-up where the liquid phase of the batch reactor could be separated into a concentrated brine solution and into a sugar-containing solution, i.e., glucose, mannose, xylose, and arabinose. Moreover, the solid phase can have other uses such as to adsorb MB, i.e., polysaccharide (amorphous and crystalline cellulose, mannan, xylan, and arabinan) and lignin production. All these possibilities can be explored within the concept of the biorefinery.

This lab-scale study could potentially be upscaled so that it could be relevant to larger-scale applications where there are limitations regarding the available amount of spruce sawdust as a byproduct from the wood industry. There are no limitations to desalination brine availability as a byproduct from the numerous desalination plants. On the other hand, there are many other uses for the spruce sawdust, as well as for the desalination brine, but they are not at an industrial scale yet. The pretreatment temperature of 160–240 °C is another limitation regarding the cost-effectiveness and the environment-friendliness of the process. Sawdust can be used directly as adsorbent, as can be noticed from these experiments, but the pretreatment under investigation enhances the adsorption capacity. Unfortunately, it increases the production cost. The suggested herein optimal temperature is 200 °C, but adsorbents with similar properties can be achieved at lower temperature, e.g., 160 °C, by increasing the pretreatment time. The limitation is to keep the logR_0_* value the same.

## 4. Conclusions

The novelty of this research is to use a harmful waste, i.e., desalination brine, for spruce sawdust pretreatment to produce an ecofriendly adsorbate. BET, SEM, FTIR, and XRD techniques were applied to investigate the structure of the novel material. According to the Freundlich isotherm model, the capacity parameter *K_F_* for desalination brine-pretreated material (240 °C, 25 min, containing 178.71 g L^−1^ NaCl and other contents) increased up to 14.8 (mg g^−1^) (L mg^−1^)^1/n^ compared to that of untreated spruce sawdust, which was 3.8 (mg g^−1^) (L mg^−1^)^1/n^; i.e., it increased approximately four times. The experimental conditions corresponding to the combined severity factor in logarithmic form logRo* equal to 1.964 provided the theoretical maximum capacity parameter *K_F_* value. The second-order kinetic capacity parameter *q_e_* for pretreated material (200 °C, 25 min, containing 98.12 g L^−1^ NaCl and other contents) increased up to 12.9 mg g^−1^ compared to that of the untreated spruce sawdust, which was 6.9 mg g^−1^; i.e., it increased approximately two times. The conditions corresponding to the combined severity factor in logarithmic form logRo* equal to −1.049 provided the theoretical maximum capacity parameter *q_e_* value. Industrial scale applications can preferably be based on the kinetic data findings because they refer to a less than 200 min adsorption period compared to the isotherms, which concern a seven-day adsorption period. Consequently, the authors suggest 200 °C, 25 min, and contents of 98.12 g L^−1^ NaCl and other contents as optimal spruce sawdust pretreatment conditions. According to these findings, autoclave desalination brine-pretreated spruce sawdust is an environmentally friendly low-cost activated carbon substitute produced within the circular economy approach.

Future work must be carried out for the MB adsorption on pretreated spruce sawdust packed in a laboratory-scale adsorption column. Most of the industrial facilities utilize adsorption columns instead of batch adsorption apparatuses. In addition, desalination brine from full-scale desalination facilities can be used instead of the simulated solution. Moreover, prior to proceeding to full-scale applications, a pilot plant batch reactor must be constructed for the spruce pretreatment. Finally, a pilot-scale batch adsorption system and a pilot-scale packed bed adsorption column must be constructed and studied. In the phase of the pilot-scale experiments, a technoeconomic analysis should be carried out, as well as a Life Cycle Assessment of the whole system. 

## Figures and Tables

**Table 1:** Adsorption capacity of methylene blue on some adsorbents.

	Freundlich		Langmuir		
Materials	*K_F_* [(mgg^−1^) (Lmg^−1^)^1/n^]	*n*	*q_m_* (mg g^−1^)	*K_L_* (L mg^−1^)	References
Wheat hull	4.13	1.78	62.50	0.04	[21]
Fig leaf activated carbon	31.91	8.86	41.70	0.37	[22]
Corn husk waste	12.20	5.55	34.56	6.90	[23]
*Humulus lupulus* stems	38.63	1.38	209.80	0.24	[24]
Lupine seed	2.28	1.35	77.48	1.88	[25]
Pumpkin seed shells	9.61	1.54	48.98	9.93	[25]
*Solanum elaeagnifolium Cavanilles*	11.06	2.19	50.61	0.20	[26]
Modified macadamia nutshells	22.21	2.00	195.95	0.07	[27]
*Rumex Crispus* L. Stem	12.25	3.05	50.00	0.14	[28]
Green pea (*Pisum sativum*) haulm	25.00	2.13	167.00	0.09	[29]
Magnetized *Azadirachta* indica sawdust	29.61	1.57	169.49	0.21	[30]
Spruce sawdust	3.80	1.40	26.0	0.084	In this work
Brine pretreated spruce sawdust	14.8	1.04	142.4	0.250	In this work

**Table 2:** Box–Behnken design of experiments.

Experiment	Temperature *T* (°C)	Time *t* (min)	Brine Concentration (NaCl in g L^−1^)
1	160	0	98.12
2	240	0	98.12
3	160	50	98.12
4	240	50	98.12
5	200	0	24.53
6	200	0	178.71
7	200	50	24.53
8	200	50	178.71
9	160	25	24.53
10	160	25	178.71
11	240	25	24.53
12	240	25	178.71
13	200	25	98.12
14	200	25	98.12
15	200	25	98.12

**Table 3:** Severity factor R_0_, combined severity factor R_0_*, and logarithm of the combined severity factor logR_0_* estimated values for spruce sawdust desalination brine pretreatment experiments.

Experiment	R_0_	R_0_*	logR_0_*
1	198	6.12 × 10^−6^	−5.213
2	5.51 × 10^4^	5.39 × 10^−2^	−1.269
3	3.28 × 10^3^	2.12 × 10^−5^	−4.674
4	7.21 × 10^5^	64.3	1.808
5	2.97 × 10^3^	1.27 × 10^−4^	−3.897
6	2.46 × 10^3^	7.60 × 10^−6^	−5.119
7	4.62 × 10^4^	0.376	−0.425
8	4.92 × 10^4^	1.39	0.142
9	1.81 × 10^3^	2.03 × 10^−5^	−4.693
10	1.75 × 10^3^	2.83 × 10^−5^	−4.548
11	3.97 × 10^5^	10.7	1.029
12	4.02 × 10^5^	92.0	1.964
13	2.84 × 10^4^	8.15 × 10^−2^	−1.089
14	2.92 × 10^4^	8.94 × 10^−2^	−1.049
15	2.73 × 10^4^	7.10 × 10^−2^	−1.149

**Table 4:** Freundlich and Langmuir isotherm models’ parameters and SEE values.

log*R*_0_*	*K_F_* [(mg g^−1^) (L mg^−1^)^1/n^]	*n*	SEE	*q_m_* (mg g^−1^)	*K_L_* (L mg^−1^)	SEE
untreated	3.80	1.40	2.67	26.0	0.084	2.58
−5.213	7.99	1.51	1.92	38.5	0.120	2.52
−1.269	10.06	1.31	1.91	55.9	0.207	2.52
−4.674	8.11	1.50	4.60	45.6	0.120	1.67
1.808	14.00	1.10	2.86	128.2	0.294	5.01
−3.897	8.10	1.50	2.62	63.9	0.027	4.54
−5.119	8.08	1.50	4.08	49.6	0.065	2.76
−0.425	9.74	1.34	4.53	85.4	0.334	5.13
0.142	9.85	1.33	4.19	99.7	0.288	2.95
−4.693	8.05	1.51	1.28	55.9	0.068	2.01
−4.548	8.05	1.51	2.88	55.7	0.042	2.81
1.029	14.77	1.03	3.15	113.9	0.354	3.48
1.964	14.80	1.04	3.72	142.4	0.250	6.49
−1.089	9.20	1.42	5.66	48.4	0.343	5.27
−1.049	9.00	1.40	4.13	58.1	0.276	5.05
−1.149	9.09	1.41	4.87	65.3	0.309	4.98

**Table 5:** Sips isotherm model’s parameters and SEE values.

log*R*_0_*	*q_m_* (mg g^−1^)	*K_L_* (L mg^−1^)	*n*	SEE
untreated	36.4	0.302	0.63	2.21
−5.213	34.6	0.160	1.25	5.22
−1.269	58.6	0.340	0.78	4.49
−4.674	43.3	0.140	1.21	3.61
1.808	70.1	0.184	0.96	6.13
−3.897	38.2	0.048	1.05	5.22
−5.119	50.9	0.060	1.04	2.91
−0.425	49.3	0.329	1.01	4.76
0.142	58.9	0.350	1.01	3.10
−4.693	30.1	0.056	1.10	2.06
−4.548	40.3	0.017	1.33	2.66
1.029	69.3	0.406	0.81	3.56
1.964	72.1	0.133	0.79	2.14
−1.089	46.4	0.375	0.75	5.23
−1.049	46.9	0.379	0.78	5.44
−1.149	46.3	0.366	0.79	5.13

**Table 6:** Lagergren (first-order) and second-order kinetic parameters and SEE values.

log*R*_0_*	*k* (min^−1^)	*q* (mg g^−1^)	SEE	*k*_2_ (g mg^−1^ min^−1^)	*q* (mg g^−1^)	SEE
untreated	0.0232	6.31	0.428	0.0071	6.90	0.198
−5.213	0.0299	7.01	0.47	0.0121	7.52	0.131
−1.269	0.0268	8.39	0.157	0.0101	11.83	0.198
−4.674	0.0297	8.97	0.455	0.0136	10.16	0.152
1.808	0.0178	2.00	0.192	0.0032	11.33	0.288
−3.897	0.0297	8.97	0.51	0.0116	11.30	0.173
−5.119	0.0298	8.90	0.446	0.0106	8.33	0.101
−0.425	0.0273	8.49	0.491	0.0071	11.83	0.192
0.142	0.0271	8.46	0.596	0.0076	12.84	0.259
−4.693	0.0298	8.97	0.394	0.0129	9.83	0.125
−4.548	0.0298	8.02	0.433	0.0141	9.99	0.109
1.029	0.0155	1.88	0.285	0.0061	12.45	0.36
1.964	0.0155	1.76	0.191	0.0039	11.05	0.374
−1.089	0.0290	8.98	0.296	0.0104	12.90	0.139
−1.049	0.0280	8.89	0.287	0.0128	12.84	0.137
−1.149	0.0283	9.13	0.275	0.0132	12.97	0.141

**Table 7:** Intraparticle diffusion kinetic model’s parameters and SEE values.

log*R*_0_*	1st Step		2nd Step		SEE
	*k_p_* (min^−1^)	*c* (mg g^−1^)	*k_p_* (min^−1^)	*c* (mg g^−1^)	
untreated	0.754	0.142	0.202	2.935	0.194
−5.213	1.510	0.485	0.206	8.443	0.371
−1.269	1.598	0.505	0.252	8.230	0.389
−4.674	1.506	0.477	0.191	7.430	0.367
1.808	0.950	0.117	0.461	3.021	0.144
−3.897	1.661	0.497	0.225	8.088	0.377
−5.119	1.598	0.413	0.208	8.533	0.317
−0.425	1.473	0.234	0.302	5.652	0.232
0.142	1.480	0.297	0.407	6.654	0.311
−4.693	1.546	0.557	0.148	7.750	0.41
−4.548	1.629	0.523	0.180	8.635	0.394
1.029	1.238	0.169	0.539	4.121	0.194
1.964	1.150	0.234	0.469	3.957	0.238
−1.089	1.631	0.338	0.286	8.641	0.251
−1.049	1.641	0.339	0.280	8.694	0.262
−1.149	1.623	0.333	0.304	8.546	0.254

**Table 8:** Assignment of spruce sawdust peaks in the FTIR spectra before and after the pretreatment.

Wavenumber [cm^−1^]			Assignment	Components
Untreated	Pretreated	Increase		
3465	3350	−115	O-H stretching of bonded hydroxyl groups	Hemicelluloses, Cellulose, Lignin
2910	2940	30	Aromatic methoxyl groups and in methyl and methylene groups of side-chain symmetric C-H stretching in	Hemicelluloses, Cellulose, Lignin
2362	2362	0	N-H stretching	Hemicelluloses, Cellulose, Lignin
1734	1710	−24	Unconjugated xylans C=O stretching	Hemicelluloses
1699	1700	1	R-OH aliphatic carboxyl groups	Lignin
1654	1651	−3	Aromatic skeletal vibration, C=O stretching in lignin, H-O-H deformation vibration of adsorbed water	Hemicelluloses, Lignin
1617	1616	−1	C=C stretching of phenol group	Hemicelluloses, Cellulose, Lignin
1576	1576	0	Aromatic skeletal vibration, C=O stretching,	Lignin
1507	1506	−1	C=C stretching of the aromatic ring and aromatical skeletal vibration in lignin	Lignin
1435	1457	22	C-H deformation in methyl and methylene	Lignin
1374	1375	1	C-H bending, C-H stretching in methylene	Hemicelluloses, Cellulose, Lignin
1335	1314	−21	CH_2_ wagging, C5 substituted aromatic units C-O stretching	Hemicelluloses, Cellulose, Lignin
1268	1278	10	C-O stretching of guaiacyl unit	Lignin
1180	1173	−7	C-H aromatic in-plane deformation	Lignin
1134	1132	−2	C-O-C stretching	Hemicelluloses, Cellulose
1042	1060	16	C-OH stretching vibration, C-O deformation	Hemicelluloses, Cellulose, Lignin
1031	1038	7	C-O stretching, C-H aromatic in-plane deformation	Cellulose, Lignin
902	867	−35	C-O-C stretching	Hemicelluloses, Cellulose
805	852	47	C-H aromatic out-of-plane bending	Lignin

## Data Availability

Data will be available on demand.
